# Differences in the Phenotype of Bacterial and Viral Sepsis—A Prospective, Multicenter, Observational Study

**DOI:** 10.3390/v17121617

**Published:** 2025-12-14

**Authors:** Fabian Perschinka, Georg Franz Lehner, Timo Mayerhöfer, Andrea Köhler, Walter Hasibeder, Christoph Krismer, Julia Killian, Dietmar Fries, Johannes Bösch, Norbert Perschinka, Peter Hohenauer, Nadine Perschinka, Anna Lisa Hackl, Michael Joannidis

**Affiliations:** 1 Department of Internal Medicine, Division of Intensive Care and Emergency Medicine, Medical University Innsbruck, 6020 Innsbruck, Austria; fabian.perschinka@i-med.ac.at (F.P.);; 2 Department of Anesthesiology and Critical Care Medicine, Hospital St. Vinzenz Zams, 6511 Zams, Austria; 3 Department of Internal Medicine, Hospital St. Vinzenz Zams, 6511 Zams, Austria; 4 Department of General and Surgical Intensive Care Medicine, Medical University Innsbruck, 6020 Innsbruck, Austria; 5 Department of Anesthesiology and Critical Care Medicine, Hospital Barmherzige Schwestern, 4910 Ried im Innkreis, Austria; 6 Department of Neurology, Hospital Barmherzige Schwestern, 4910 Ried im Innkreis, Austria

**Keywords:** bacterial infection, critically ill, intensive care unit, sepsis, SEPSIS-3, SOFA, viral infection

## Abstract

Sepsis is defined as a dysregulated host response to an infection, leading to life-threatening organ dysfunction. While sepsis is most commonly the result of a bacterial infection, it may also be caused by viral pathogens. The aim of this study was to describe differences in organ dysfunction patterns and inflammatory markers between bacterial and viral sepsis. In this prospective multicenter cohort study, adults meeting SEPSIS-3 criteria were recruited from four Austrian ICUs between 1 August 2021 and 1 April 2024, excluding those who were immunocompromised within the preceding 12 months. Ninety patients were enrolled, of whom 57 had bacterial and 33 viral sepsis. Inflammatory markers, including IL-6 and PCT, were higher at ICU admission in bacterial sepsis. Adjusted linear regression confirmed bacterial etiology as the only significant predictor of higher 48 h peak IL-6 and PCT values. Patients with viral sepsis typically fulfilled SEPSIS-3 criteria through respiratory and cardiovascular SOFA components, while other organ dysfunctions were less frequent. Significant differences in the phenotype of bacterial and viral sepsis were observed, characterized by distinct inflammatory profiles and differing patterns of organ dysfunction. These findings may support the improved differentiation of bacterial and viral etiologies in sepsis.

## 1. Introduction

Sepsis causes millions of deaths worldwide every year. According to the SEPSIS-3 definition, sepsis is a life-threatening organ dysfunction resulting from a dysregulated host response to infection. The presence of organ dysfunction distinguishes sepsis from an uncomplicated infection [1].

Sepsis is a complex syndrome influenced by both pathogen- and host-related factors. It is primarily caused by bacterial infections, with respiratory infections being by far the most common source [2]. Studies conducted before the COVID-19 pandemic indicated that fewer than 5% of intensive care unit (ICU) patients had a viral pathogen as the causative agent [3]. However, viruses are likely underestimated as causes of sepsis and may contribute substantially to cases of culture-negative sepsis [4]. In recent years, particularly during the COVID-19 pandemic, viral infections have been recognized as capable of causing severe disease requiring ICU treatment [5] and meeting the SEPSIS-3 criteria for sepsis. Importantly, the SEPSIS-3 definition is pathogen-independent, meaning that the same diagnostic criteria apply to viral infections [6]. Nevertheless, retrospective analyses of common inflammatory markers, including interleukin 6 (IL-6), interleukin 8 (IL-8), procalcitonin (PCT), tumor necrosis factor (TNF) and c-reactive protein (CRP), have demonstrated significant differences in the inflammatory profiles between bacterial and viral infections [7,8].

Both bacterial and viral pathogens that lead to sepsis most commonly cause respiratory tract infections. In critically ill septic patients, multiple organ systems are often affected [9]. The mechanisms underlying organ dysfunction in sepsis are diverse, ranging from circulatory abnormalities and endothelial dysfunction to cellular and mitochondrial dysfunction. Furthermore, organ injury in one system can propagate dysfunction in others through inter-organ crosstalk [9,10].

We hypothesized that bacterial sepsis is associated with higher levels of inflammatory markers than viral sepsis, although both meet the SEPSIS-3 criteria. Additionally, we hypothesized that the two etiologies differ in the specific organ systems contributing to a Sequential Organ Failure Assessment (SOFA) score increase of ≥2.

The aim of this study was to characterize distinct profiles of organ dysfunction and inflammatory markers in bacterial and viral sepsis among patients meeting SEPSIS-3 criteria.

## 2. Materials and Methods

### 2.1. Patients

This was a prospective, multicenter, observational study. Patients from four ICUs were enrolled, two of which were part of the Medical University of Innsbruck (16 and 11 multipurpose ICU beds) and two from secondary hospitals (7 and 7 multipurpose ICU beds). The recruitment period extended from 1 August 2021, to 1 April 2024. The Hospital Barmherzige Schwestern Ried im Innkreis joined the study later (January 2022). Patient recruitment was conducted consecutively in parallel across all participating ICUs. Patients were included if they fulfilled the sepsis criteria according to SEPSIS-3 [1] and had a planned ICU stay of at least 48 h. For assignment to the bacterial sepsis group, the pathogen had to be identified by at least one positive aerobic or anaerobic blood culture, a positive PCR-based test, detection of a bacterial antigen, or a radiologically confirmed site of infection strongly suggestive of bacterial infection. Patients were assigned to the viral sepsis group if a virus was detected at least once by an antigen-based test or PCR-based test of nasal/bronchial swabs or blood samples.

All patients underwent comprehensive microbiological diagnostics to rule out co-infections, including aerobic and anaerobic blood cultures. In cases of suspected respiratory infection, sputum, nasopharyngeal swabs, tracheal aspirates, or bronchoalveolar lavage (BAL) samples were additionally analyzed. When the suspected site of infection was accessible, site-specific swabs were collected (e.g., dermal or intestinal samples obtained during surgery). Only patients with a confirmed viral or bacterial infection at the time of inclusion were considered, while patients with co-infections were excluded. If no definitive site of infection was identified, a whole-body CT scan was performed to locate potential sites of infection. Patients were excluded if they exhibited immunosuppression due to comorbidities, medication, or chemotherapy/radiotherapy within the last 12 months before screening. A flowchart of inclusion is presented in ESM Figure S1.

### 2.2. Definitions and Data Collection

The day of ICU admission was defined as baseline (day 0). Laboratory parameters and co-infections were recorded for seven days or until ICU discharge if transferred to a regular ward earlier. All other ICU-related data were collected for the entire ICU stay.

IL-6 and PCT were measured in Li-heparin plasma using electrochemiluminescence immunoassays, with lower detection limits of 3.5 ng/L and 0.06 µg/L, respectively. CRP and ferritin were determined in Li-heparin plasma using particle-enhanced turbidimetric immunoassays, with lower detection limits of 0.06 mg/dL and 8 µg/L, respectively.

Acute kidney injury (AKI) was defined according to the Kidney Disease: Improving Global Outcomes (KDIGO) guidelines [11]. The SOFA score and the Simplified Acute Physiology Score 3 (SAPS 3) were used to grade disease severity. Scores were calculated on the day of ICU admission and daily for the subsequent seven days. Both scores were defined as missing if one or more required laboratory parameters were unavailable.

Data were recorded until death or hospital discharge, whichever occurred first. All participating centers used an electronic case report form based on the REDCap electronic data capture system [12,13].

### 2.3. Statistical Analysis

The sample size was determined based on the results of a previously published retrospective pilot study [7]. Based on these data, a minimum of 80 patients was required to achieve 90% power at a two-sided significance level of α = 0.05. To account for an anticipated dropout rate of approximately 10%, the target sample size was increased to 90 patients.

The study hypothesis, as well as all primary and secondary endpoints, were determined prior to patient recruitment and data collection. The primary endpoints were defined as the maximum IL-6 and PCT levels reached within 48 h after ICU admission. Secondary exploratory endpoints included (1) the distribution of SOFA points across individual organ system components, (2) the need for invasive mechanical ventilation, and (3) mortality rates. Predefined subgroup analyses compared (a) *SARS*-*CoV*-2 with other viral etiologies and (b) bacterial versus viral sepsis, restricted to patients with a respiratory focus.

Continuous variables were presented as medians with interquartile ranges (IQRs), while categorical variables were described as counts and corresponding percentages. Normal distribution was assessed using the Shapiro–Wilk test. For non-normally distributed data, the Mann–Whitney U test was applied, and for normally distributed data, Student’s t-test was used. Categorical variables were analyzed using the Chi-squared test. In longitudinal analyses, patients who died or were discharged before completing the seven-day observation period were censored at the time of death or discharge, and missing data were not imputed.

The primary endpoints were analyzed using an unadjusted Mann–Whitney U test. To adjust for potential bias, we additionally performed an adjusted analysis using a linear logistic regression model including bacterial etiology, SAPS 3 at admission, sex, site of infection, and age as covariates. Because IL-6 and PCT were not normally distributed, their values were log-transformed for this adjusted analysis.

To determine whether the trajectories of inflammatory markers differed independently between bacterial and viral sepsis, we performed adjusted analyses using linear mixed-effects models. The dependent variables were the repeated measures of IL-6, PCT, CRP, and ferritin. Fixed effects included infection etiology (bacterial or viral), sex, age, site of infection, all recorded comorbidities, and baseline disease severity assessed by the SAPS 3 score. Additionally, a separate adjusted model—with the same set of covariates—was constructed to investigate whether infection etiology was associated with the number of organ dysfunctions.

To account for the fact that most viral cases involved a pulmonary infection, analyses were repeated in the subgroup of patients with a respiratory focus. Additionally, differences between *SARS*-*CoV*-2 and other viral pathogens were analyzed in a separate subgroup analysis.

For statistical analyses, SPSS software (version 29; IBM Corp., Armonk, NY, USA) was used. All tests were two-sided, and a *p*-value < 0.05 was considered statistically significant.

## 3. Results

In total, 90 patients were included in this analysis, of whom 57 had bacterial sepsis and 33 were diagnosed with viral sepsis. Both groups were of similar median age and predominantly male (66.7% vs. 75.8%; *p* < 0.001), although patients with bacterial sepsis exhibited higher SAPS 3 and SOFA scores at ICU admission. All patients in the viral sepsis group had a respiratory site of infection, whereas most patients in the bacterial sepsis group were diagnosed with respiratory (26.8%), abdominal (25.0%), or urogenital (21.4%) infections. Detected pathogens are listed in ESM Table S1. Most patients in both groups (91.2% vs. 84.8%; *p* = 0.353) required vasopressors at ICU admission.

Detailed baseline characteristics and comorbidities are presented in Table 1.

Viral sepsis patients more frequently required IMV and extracorporeal membrane oxygenation (ECMO), whereas AKI occurred significantly more often in bacterial sepsis. In our cohort, the incidence of AKI was approximately 76% in the bacterial sepsis group and 45% in the viral sepsis group (*p* = 0.004). The majority of patients in both groups were treated with corticosteroids. Dexamethasone was predominantly used in viral sepsis, while hydrocortisone was more commonly administered in bacterial sepsis. Therapies and complications during the ICU stay are summarized in ESM Table S2, and details on administered corticosteroids are provided in ESM Table S3.

Both ICU and hospital mortality were higher in the bacterial sepsis group (ICU: 22.8% vs. 12.1%; *p* = 0.212; hospital: 26.3% vs. 15.2%; *p* = 0.220). The standardized mortality ratios (SMR; observed-to-expected mortality ratio) based on SOFA and SAPS 3 scores were 0.69 and 0.52, respectively, in bacterial sepsis, compared to 0.56 and 0.40, respectively, in viral sepsis.

### 3.1. Inflammatory Markers

During the first days in the ICU, IL-6 levels were significantly higher in the bacterial sepsis group compared to the viral sepsis group. The highest median values were observed at ICU admission (bacterial: 2740.50 ng/L [IQR: 626.00–30,785.00]; viral: 165.00 ng/L [IQR: 39.10–460.20]; *p* < 0.001), followed by a subsequent decline that was more pronounced in bacterial sepsis. The highest median IL-6 value within the first 48 h of ICU stay was significantly higher in bacterial sepsis than in viral sepsis (1848.00 ng/L [382.00–20,961.00] vs. 188.00 ng/L [84.90–540.00]; *p* < 0.001). In the linear regression model evaluating factors associated with the maximum median IL-6 concentration within 48 h of ICU admission, bacterial etiology was the only variable that remained statistically significant (*p* < 0.001). SAPS 3 at admission showed a non-significant trend toward an association (*p* = 0.071), while age, sex, and site of infection were not associated (ESM Table S4).

A similar pattern was observed for PCT levels: patients with bacterial sepsis exhibited significantly higher levels from baseline through day seven. The initial median value (16.52 µg/L [IQR: 2.84–57.05]) declined steadily and remained significantly higher by day seven (bacterial: 1.66 µg/L [IQR: 0.53–2.70]; viral: 0.13 µg/L [IQR: 0.05–0.22]; *p* < 0.001). Within 48 h of ICU admission, PCT levels reached higher levels in bacterial sepsis (17.10 µg/L [8.55–88.90] vs. 0.44 µg/L [0.22–2.05]; *p* < 0.001). In the corresponding linear regression assessing determinants of the 48 h peak PCT concentration, bacterial etiology again emerged as the only statistically significant factor (*p* < 0.001) (ESM Table S4).

**Figure 1:**
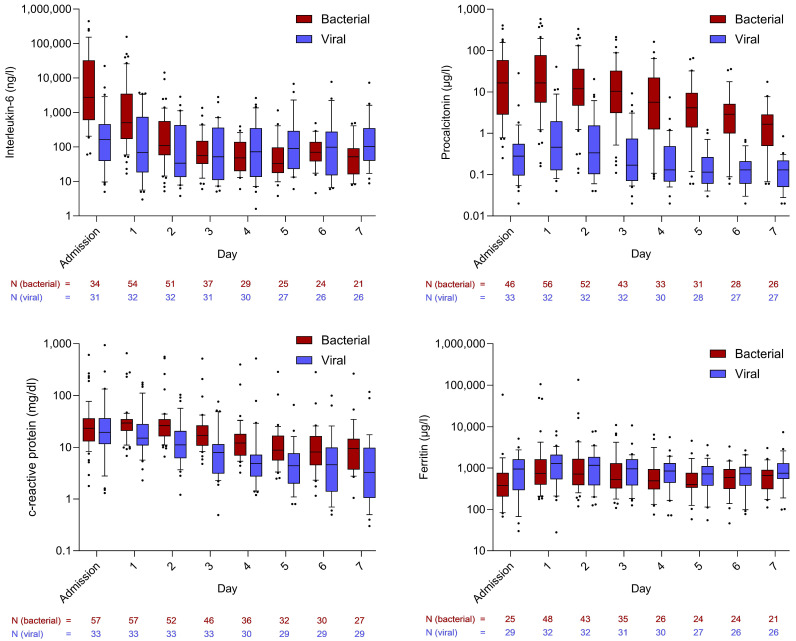
Courses of inflammatory markers in comparison between bacterial and viral sepsis patients. Whiskers extend to cover all values from the 1st to the 9th percentile. Dots plotted above or below the whiskers represent individual patient values outside this range and are therefore considered outliers.

CRP levels were also higher in bacterial sepsis, although the difference did not reach statistical significance. In contrast, ferritin levels were higher in viral sepsis. The courses of all four inflammatory parameters are shown in Figure 1.

**Figure 2:**
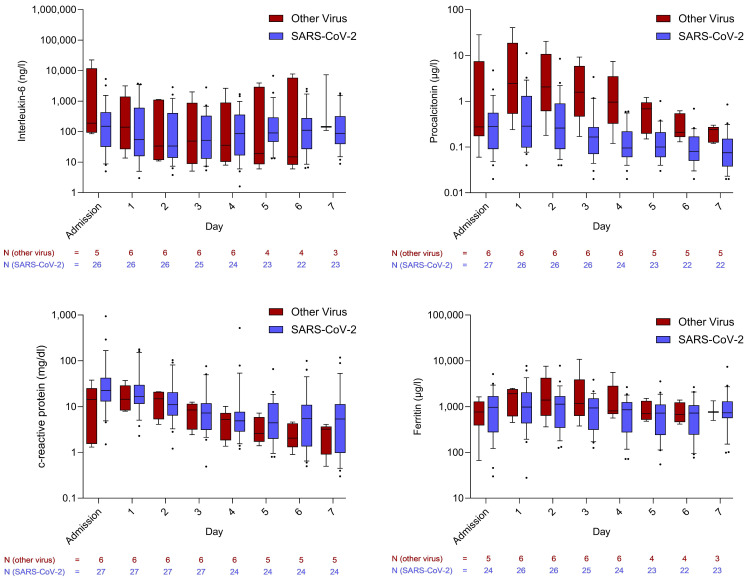
Courses of inflammatory markers in comparison between infections caused by *SARS*-*CoV*-2 and other viruses. Whiskers extend to cover all values from the 1st to the 9th percentile. Dots plotted above or below the whiskers represent individual patient values outside this range and are therefore considered outliers.

To explore differences among viral pathogens, a subgroup analysis compared *SARS*-*CoV*-2 with other viruses. Comparable trends in inflammatory markers were observed between the two groups. However, PCT levels were significantly lower in patients with *SARS*-*CoV*-2 infection compared to those with other viral infections (Figure 2).

Approximately one-third of the patients with *SARS*-*CoV*-2 infection had received prior vaccination. No significant differences were observed in PCT or CRP levels between vaccinated and unvaccinated individuals. IL-6 levels showed a non-significant trend toward lower values in vaccinated patients. Nonetheless, both vaccinated and unvaccinated *SARS*-*CoV*-2 patients exhibited markedly lower IL-6 and PCT levels compared to those with bacterial sepsis. Ferritin concentrations were lower in vaccinated patients than in unvaccinated *SARS*-*CoV*-2 patients. The trajectories of these inflammatory markers are shown in ESM Figure S2.

### 3.2. Adjusted Linear Mixed-Effects Models

In adjusted linear mixed-effects model analyses, bacterial etiology was independently associated with higher IL-6 and PCT levels over time, but not with CRP. IL-6 levels were also influenced by sex and age, whereas ferritin levels were associated with the site of infection. Baseline disease severity (SAPS 3) at ICU admission showed a significant association only with IL-6 (ESM Table S5). In a separate adjusted model, bacterial sepsis, male sex, and a higher SAPS 3 score at ICU admission were each independently linked to a greater number of organ dysfunctions (ESM Table S6).

### 3.3. Differences in Fulfilling SEPSIS-3 Criteria

Significantly more viral sepsis patients scored 3 or 4 points in the respiratory component of the SOFA score at ICU admission (81.3% vs. 42.2%; *p* < 0.001). Four points due to hemodynamic support (norepinephrine > 0.1 µg/kg/min) were predominantly observed in the bacterial sepsis group, whereas 3 points (norepinephrine ≤ 0.1 µg/kg/min) were more common in viral sepsis (ESM Table S7). In the other SOFA components, two, three or four points were predominantly observed in bacterial sepsis patients. Almost all viral sepsis patients met SEPSIS-3 criteria due to respiratory or cardiovascular dysfunction, and over 90% scored 0 points in the hepatic and coagulation components; approximately two-thirds had no points in the renal SOFA. The estimated pre-sedation GCS was 14 or 15 in all cases, and therefore no GCS-related SOFA points were assigned.

**Figure 3:**
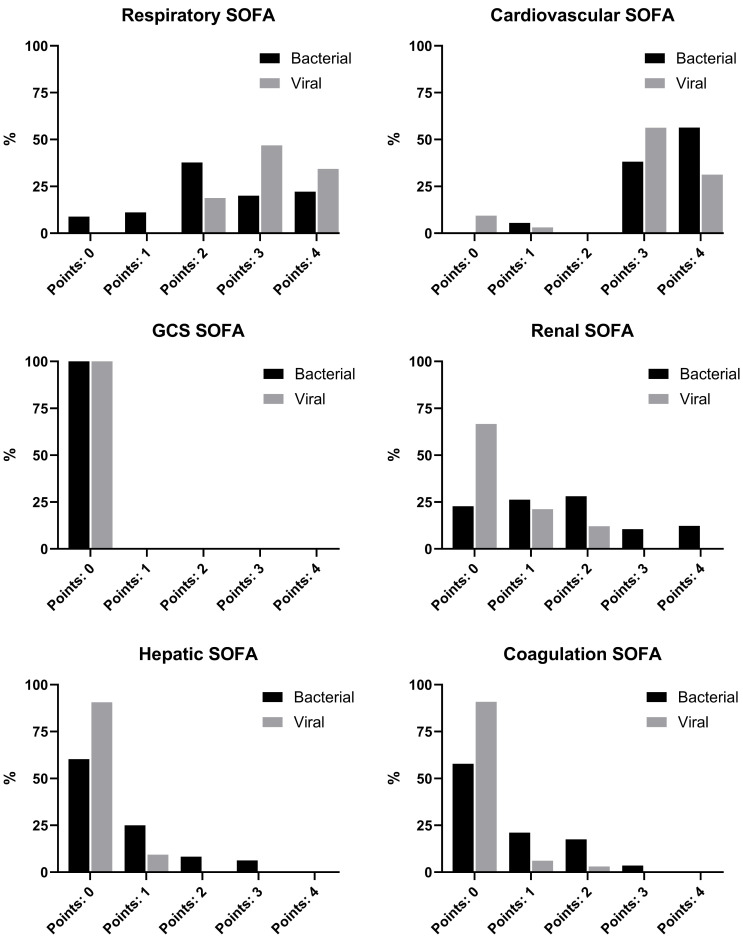
SOFA scores at ICU admission.

The detailed SOFA component distribution at ICU admission is shown in Figure 3.

When excluding the points of cardiovascular and respiratory SOFA at ICU admission, a minority of viral sepsis patients reached the threshold of two points, whereas the rates were significantly higher in the bacterial sepsis group. This difference persisted throughout the ICU stay (ESM Table S8).

### 3.4. Analysis Restricted to Patients with a Respiratory Focus

The baseline characteristics, therapies, and complications of patients with a respiratory focus are presented in ESM Tables S9 and S10. Trajectories of inflammatory markers in this subgroup were comparable to those seen in the overall cohort (ESM Figure S3). When analyzing the highest IL-6 and PCT values within 48 h of ICU admission restricted to patients with a respiratory focus, results remained consistent with the findings in the overall cohort. Both maximum median IL-6 levels (2513 ng/L [626.00–24,245.00] vs. 188.00 ng/L [84.90–540.00]; *p* < 0.001) as well as PCT levels (17.80 µg/L [8.72–57.05] vs. 0.44 µg/L [0.22–2.05]; *p* < 0.001) were significantly higher in patients with bacterial sepsis.

In adjusted linear mixed-effects models restricted to patients with a respiratory focus, bacterial sepsis remained associated with IL-6 (*p* = 0.006), PCT (*p* = 0.001), and CRP (*p* = 0.037) levels, while other covariates showed no consistent associations across the three markers over the observation period. No association was found between bacterial sepsis and ferritin (ESM Table S11).

A similar pattern was observed regarding organ dysfunction and fulfillment of the SEPSIS-3 criteria: when excluding respiratory and cardiovascular SOFA components, two-thirds of bacterial sepsis patients still had a SOFA ≥ 2 at ICU admission, compared to 18.2% in the viral sepsis group. By day seven, these proportions had declined to approximately 45% (bacterial sepsis) and 14% (viral sepsis). Detailed subgroup results are shown in ESM Table S12.

## 4. Discussion

In this prospective, observational, multicenter cohort study of critically ill sepsis patients meeting SEPSIS-3 criteria, we observed notable differences in the phenotype of bacterial and viral sepsis. Although all patients fulfilled the SEPSIS-3 criteria, the phenotype of sepsis caused by viral pathogens was associated with lower levels of inflammatory parameters from admission through the first 7 days of ICU stay, as well as a lower frequency of multiple organ dysfunctions. Disease severity appeared to be moderately lower in patients with viral sepsis, mainly involving respiratory and cardiovascular impairment.

The inflammatory parameters—particularly IL-6 and PCT—were substantially higher at ICU admission among patients with bacterial infection. Whereas IL-6 levels declined rapidly over the following 3 days, the difference in PCT levels between the groups persisted during the first week of ICU stay. The immune response is mediated by the recognition of pathogen-associated molecular patterns (PAMPs) via Toll-like receptors (TLRs), which differ between bacterial and viral pathogens [14]. Bacteria and viruses are known to carry distinct PAMPs. Viral PAMPs stimulate TLRs in a manner that differs from that of bacterial PAMPs; however, it is notable that some TLRs are capable of being activated by both bacterial and viral PAMPs [15]. The observed data suggest that bacterial infections may elicit a more pronounced cytokine response than viral infections in critically ill patients. This assumption is confirmed by the adjusted linear regression analyses of the primary endpoints, in which bacterial etiology was the only variable strongly associated with higher 48 h peak IL-6 and PCT values, and further supported by the adjusted linear mixed-effects models, which demonstrated significant associations between bacterial sepsis and higher levels of IL-6 and PCT over 7 days. Nevertheless, this finding should be interpreted cautiously given the observational design. The observed decline in cytokine levels in bacterial sepsis might reflect effective antimicrobial therapy and infection control measures. As corticosteroids were used in most patients, and at similar rates overall, their potential confounding influence cannot be excluded but is likely minimal. The differences in the rates of corticosteroid administration—dexamethasone predominantly in the viral sepsis group, hydrocortisone primarily in the bacterial sepsis group—were attributed to divergent guidelines.

Our findings are consistent with previous retrospective analyses from our group showing lower inflammatory markers in COVID-19–associated sepsis compared to bacterial sepsis [7]. In this expanded analysis, which also included other viral etiologies (approximately 20%), similar trends were observed, with PCT levels lower in *SARS*-*CoV*-2 patients compared to the other viruses. Across viral etiologies (‘other viruses’ and ‘*SARS*-*CoV*-2’), inflammatory markers were consistently lower than in bacterial sepsis. The role of cytokines in the pathophysiology and progression of sepsis has long been recognized. They are key mediators of inflammation and cellular differentiation and exert reciprocal regulatory effects on one another [16]. However, evidence remains inconsistent regarding whether initial IL-6, PCT, or CRP levels are associated with 28-day mortality or predictive of outcome [17,18,19,20]. The mortality rates observed in our analysis align with published data [21,22], despite the significantly greater inflammation in our group. The significant difference in mortality between the bacterial and viral groups appears to result primarily from a lower mortality rate in the viral sepsis group, as demonstrated by the lower SMR. This observation supports the notion of differing clinical phenotypes, which merits further investigation.

Our findings are largely consistent with those reported by Cao et al. in their retrospective analysis [23] comparing bacterial and viral sepsis. In that study, both PCT and CRP levels were significantly lower in patients with viral sepsis at ICU admission, a pattern that aligns closely with the results of our cohort. By extending the observation period to seven days, our study confirms these early differences in inflammatory markers and provides additional insight into their temporal evolution during the ICU stay. However, our results differ from those of Cao et al. regarding mortality outcomes: we observed higher mortality among patients with bacterial sepsis. This discrepancy may be attributed to the lower SMR observed in the viral sepsis group in our cohort, as well as the higher baseline disease severity of our bacterial sepsis group compared with that reported by Cao et al. Another potential factor contributing to this divergence is the exclusion of immunocompromised patients in our study, whereas immunosuppression was identified by Cao et al. as a significant risk factor for ICU mortality in viral sepsis.

During ICU stay, differences were also observed in the occurrence of complications and the need for interventions. AKI is a common complication in sepsis, with up to 47% of AKIs in critically ill patients (sepsis associated-AKI; SA-AKI) [24,25]. SA-AKI arises from multiple mechanisms, including systemic and renal inflammation [26]. In our cohort, AKI was more frequent in bacterial sepsis, which could relate to the higher burden of systemic inflammation, contributing to renal inflammation [26]. In our viral sepsis group, the incidence of AKI was comparable to rates reported in other investigations. For instance, a large-scale study involving roughly 5500 ICU patients with COVID-19 or influenza found an overall AKI incidence of 43.5% (37.8% in COVID-19 and 53.7% in influenza patients) [27]. Another study similarly reported AKI rates within this range [22]. Nevertheless, given that our study was conducted several years into the COVID-19 pandemic, during which widespread vaccination campaigns were implemented in Austria, we cannot exclude the possibility that vaccination could have mitigated the risk or severity of AKI development. The delayed onset of AKI among patients with viral sepsis might suggest a greater contribution of ICU-acquired factors rather than an initial inflammatory insult.

Most patients with viral sepsis fulfilled SEPSIS-3 criteria through respiratory or cardiovascular SOFA components, while renal or hepatic dysfunctions as well as platelet reduction leading to points in the SOFA score were less common. Earlier analyses, such as that by Vincent et al. in 1998, demonstrated an uneven distribution of SOFA points across organ systems in septic patients [28]. Similarly, an analysis of control arms from the PROWESS and sPLA2 sepsis trials found that 76.3% of patients had dysfunction in at least two organ systems, and 42.8% in three or more [29]. In our study, dysfunction affecting three or more organs was more frequent in bacterial sepsis, whereas in viral sepsis, organ failure was largely confined to respiratory and cardiovascular systems. While respiratory deterioration clearly reflects viral pulmonary infection, cardiovascular impairment is likely multifactorial. A proportion of patients likely experienced hypovolemia [30]. Hypovolemia has been described as a frequent finding during the early stages of the disease, whereas the subsequent management of ARDS typically involves a restrictive fluid strategy [30,31]. Moreover, the use of low-dose vasopressors may reflect supportive measures during sedative therapy rather than refractory shock. The overall reduction in inflammatory markers argues against substantial cytokine-mediated cardiovascular dysfunction in viral sepsis.

At ICU admission, renal, hepatic, and coagulation SOFA components also differed between groups. The lower frequency of these organ dysfunctions in viral sepsis may be related to the differing profiles of inflammatory markers. Since systemic inflammation can mediate organ damage through inter-organ crosstalk [32], the observed divergence in inflammatory marker kinetics could contribute to the distinct clinical phenotypes. Nevertheless, given that organ dysfunction is a defining element of sepsis in the SEPSIS-3 criteria, it may be questioned whether the viral sepsis pattern—characterized by low inflammatory markers and infrequent organ dysfunction—truly represents sepsis, despite formally meeting the SOFA threshold [1].

### Strengths and Limitations

This observational study has some limitations. Although patients with co-infection were excluded according to the study protocol, co-infection at the time of inclusion cannot be completely ruled out despite a very precise work-up due to the diagnostic limitations inherent to clinical practice. However, in 94% of cases, the specific pathogen causing sepsis was successfully identified in the absence of simultaneous viral or bacterial pathogens. Further, only viral infections of respiratory origin were included, limiting generalizability to other viral sepsis entities. Additionally, genotypic information on *SARS*-*CoV*-2 and *Influenza virus* variants was not available. A major strength is the multicenter prospective recruitment of patients, with a balanced ratio between tertiary and secondary hospitals. Furthermore, all patients were observed for 7 days (or until ICU discharge, if transferred to a regular ward earlier) and blood was drawn daily, which enabled us to show inflammatory parameters and SOFA scores as 7-day trajectories..

## 5. Conclusions

Although all patients in both groups fulfilled SEPSIS-3 criteria, this analysis revealed distinct patterns in inflammatory markers and organ dysfunction between bacterial and viral sepsis. The findings suggest that bacterial sepsis is associated with stronger systemic inflammation and broader organ involvement, while viral sepsis presents as a less inflammatory, more organ-specific phenotype. These divergent inflammatory patterns may ultimately support more refined diagnostic and therapeutic stratification of sepsis in the future.

## Figures and Tables

**Table 1:** Baseline characteristics of the bacterial sepsis group and the viral sepsis group.

		Bacterial Sepsis (*n* = 57)	Viral Sepsis (*n* = 33)	*p*
Age °		67 (61–76)	66 (51–71)	0.082
Sex (male) *		38 (66.7%)	25 (75.8%)	<0.001
BMI °		25.40 (23.59–29.39)	28.06 (23.20–30.86)	0.340
HBA1c% °		5.80 (5.50–6.40)	6.00 (5.50–6.40)	0.582
SOFA at ICU admission °		9 (8–10)	7 (6–8)	<0.001
SAPS 3 at ICU admission °		64 (56–72)	57 (52–67)	0.021
Hospital admission due to sepsis symptoms *		33 (57.9%)	2 (6.7%)	<0.001
Proven/suspected site of infection *	Respiratory	15 (26.8%)	33 (100%)	<0.001
	Abdominal	14 (25.0%)	0	
	Urogenital	12 (21.4%)	0	
	Dermal	7 (12.5%)	0	
	Other	8 (14.3%)	0	
Risk factor *	Trauma	0	1 (3.0%)	0.005
	Surgery	15 (26.3%)	0	
	Sepsis in PMH	4 (7.0%)	1 (3.0%)	
	No risk factor	38 (66.7%)	31 (93.9%)	
SIRS fulfilled at ICU admission *		43 (75.4%)	13 (40.6%)	0.001
Vasopressors at ICU admission *		52 (91.2%)	28 (84.8%)	0.353
Norepinephrine dose at ICU admission (µg/kg/min) °		0.16 (0.09–0.32)	0.07 (0.04–0.15)	0.001
Vasopressin dose at ICU admission (U/h) °		1.60 (1.60–2.40)	1.60 (1.60–2.40)	0.894
**Inflammatory markers at ICU admission**				
Interleukin-6 (ng/L) °		2740.50 (626.00–30,785.00)	165.00 (39.10–460.20)	<0.001
Procalcitonin (µg/L) °		16.52 (2.84–57.05)	0.28 (0.10–0.55)	<0.001
C-reactive protein (mg/dL) °		23.20 (13.27–34.43)	19.50 (12.34–34.90)	0.482
Ferritin (µg/L) °		378.10 (105.00–766.00)	951.00 (325.00–1640.00)	0.042
**Comorbidities**				
Hypertension *		34 (59.6%)	11 (33.3%)	0.016
Coronary artery disease *		23 (40.4%)	8 (24.2%)	0.121
Atrial fibrillation *		15 (26.3%)	7 (21.2%)	0.587
COPD *		9 (15.8%)	1 (3.0%)	0.063
Diabetes mellitus type I *		1 (1.8%)	0	0.444
Diabetes mellitus type II *		17 (29.8%)	7 (21.2%)	0.373
Neurologic comorbidity *		1 (1.8%)	5 (15.2%)	0.014
Hepatic comorbidity *		7 (12.3%)	0	0.036
Pulmonary comorbidity *		4 (7.0%)	4 (12.1%)	0.412
Chronic kidney failure *		8 (14.0%)	9 (27.3%)	0.122
**Outcome**				
ICU mortality *		13 (22.8%)	4 (12.1%)	0.212
Hospital mortality *		15 (26.3%)	5 (15.2%)	0.220
28-Day mortality *		14 (24.6%)	4 (12.1%)	0.155
Standardized mortality rate (SOFA)		0.69	0.56	-
Standardized mortality rate (SAPS 3)		0.52	0.40	-

* *n* (%); ° median (IQR). ICU—intensive care unit; SOFA—sequential organ failure assessment; SAPS 3—simplified acute physiology score; PMH—past medical history; SIRS—systemic inflammatory response syndrome; COPD—chronic obstructive pulmonary disease. SIRS criteria were fulfilled ≥2 points.

## Data Availability

The datasets used and/or analyzed during the current study are available from the corresponding author on reasonable request.

## Supplementary Materials

The following supporting information can be downloaded at: https://www.mdpi.com/article/10.3390/v17121617/s1, ESM Figure S1: Flowchart of patient recruitment; ESM Table S1: Diagnosed pathogens at ICU admission suspected causing the sepsis; ESM Table S2: Therapy and complications of bacterial and viral sepsis patients; ESM Table S3: Corticosteroids administered in bacterial and viral sepsis patients; ESM Table S4: Adjusted linear regression analysis of factors associated with the maximum IL-6 and PCT levels within 48 h after ICU admission; ESM Figure S2: Course of inflammatory markers in in SARS-CoV-2-positive patients: comparison between vaccinated and unvaccinated groups; ESM Table S5: Adjusted linear mixed-effects models of associations between infection etiology and inflammatory marker trajectories over time; ESM Table S6: Adjusted linear mixed-effects model of associations between infection etiology and number of organ dysfunctions; ESM Table S7: Doses of norepinephrine and vasopressin per day; ESM Table S8: Rate of patients fulfilling SEPSIS-3 criteria for sepsis excluding respiratory and cardiovascular SOFA; ESM Table S9: Baseline characteristics of the bacterial sepsis group and the viral sepsis group restricted to patients with respiratory infection; ESM Table S10: Therapy and Outcome of bacterial and viral sepsis patients restricted to patients with respiratory infection; ESM Figure S3: Course of inflammatory markers in comparison between bacterial and viral sepsis patients restricted to patients with respiratory infection; ESM Table S11: Adjusted linear mixed-effects models of associations between infection etiology and inflammatory marker trajectories over time restricted to patients with respiratory infection; ESM Table S12: Rate of patients fulfilling SEPSIS-3 criteria for sepsis excluding respiratory and cardiovascular SOFA restricted to patients with respiratory infection.
