# Supplementary material for: Differences in the Phenotype of Bacterial and Viral Sepsis—A Prospective, Multicenter, Observational Study
Source: Viruses. 2025 Dec 14;17(12):1617. doi: 10.3390/v17121617 (PMC12737688; doi:10.3390/v17121617)
Supplement: Supplementary file 1 [file viruses-17-01617-s001.zip › viruses-3974781-supplementary.pdf]

Electronic Supplemental Material

Differences in the Phenotype of Bacterial and Viral Sepsis — A Prospective, Multicenter, Observational Study

Fabian Perschinka[1], Georg Franz Lehner[1], Timo Mayerhöfer[1], Andrea Köhler[1], Walter Hasibeder[2], Christoph Krismer[3], Julia Killian[2], Dietmar Fries[4], Johannes Bösch[4], Norbert Perschinka[5], Peter Hohenauer[5], Nadine Perschinka[5], Anna Lisa Hackl[6], Michael Joannidis[1,*]

1 Department of Internal Medicine, Division of Intensive Care and Emergency Medicine, Medical University Innsbruck, 6020 Innsbruck, Austria; fabian.perschinka@i-med.ac.at (F.P.);

2 Department of Anesthesiology and Critical Care Medicine, Hospital St. Vinzenz Zams, 6511 Zams, Austria;

3 Department of Internal Medicine, Hospital St. Vinzenz Zams, 6511 Zams, Austria;

4 Department of General and Surgical Intensive Care Medicine, Medical University Innsbruck, 6020 Innsbruck, Austria;

5 Department of Anesthesiology and Critical Care Medicine, Hospital Barmherzige Schwestern, 4910 Ried im Innkreis, Austria;

6 Department of Neurology, Hospital Barmherzige Schwestern, 4910 Ried im Innkreis, Austria;

* Correspondence: michael.joannidis@i-med.ac.at; Tel.: +43-512-504-24180

ESM Figure S1: Flowchart of patient recruitment

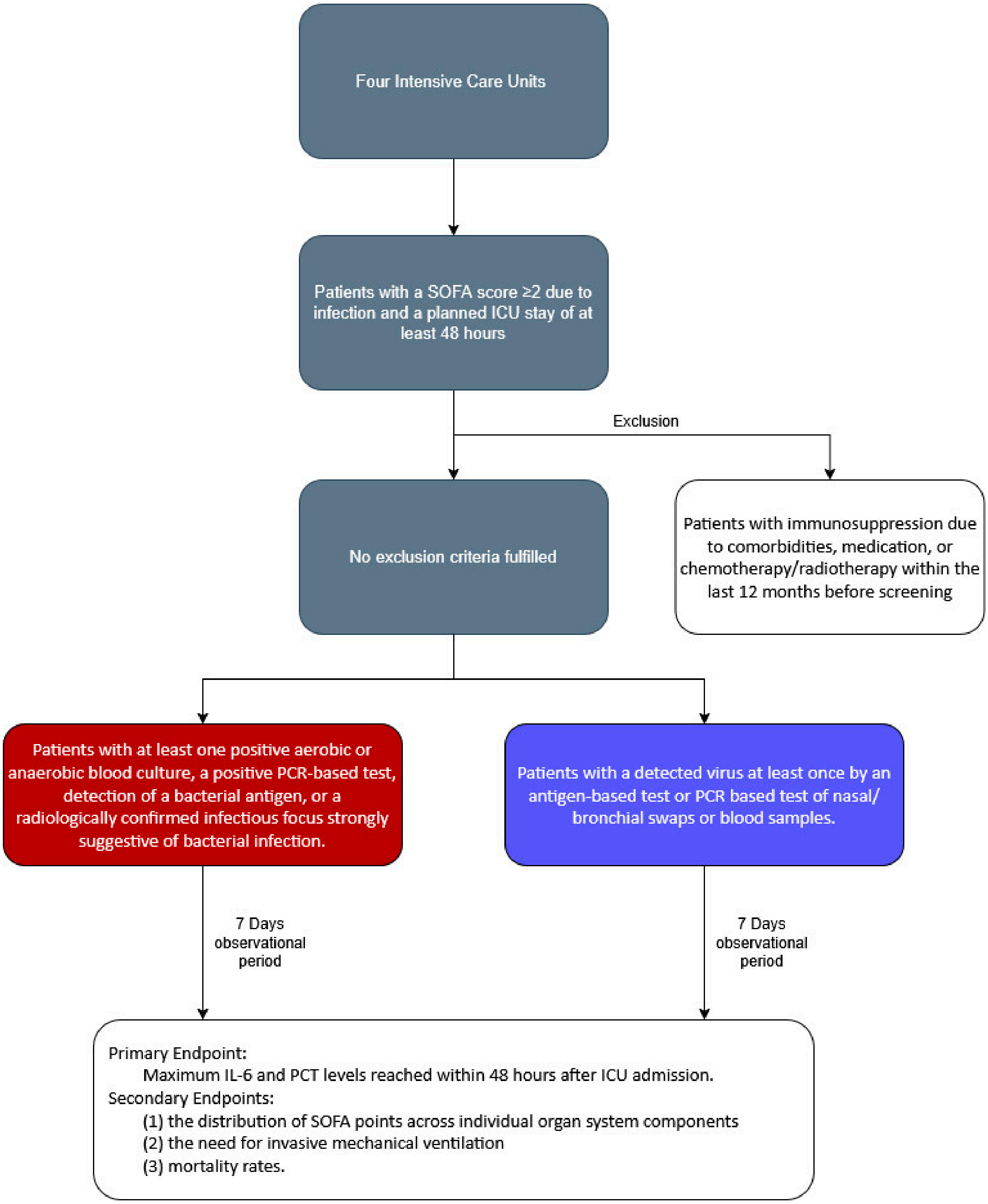

ESM Table S1: Diagnosed pathogens at ICU admission suspected causing the sepsis

Bacterial	Viral
1x Actinomyces turicensis	*1x Enterovirus*
1x Atopobium parvulum	*1x Human Rhinovirus*
1x Bacteroides fragilis	*4x Influenza A*
1x Desulfovibrio desulfuricans	*1x RSV*
1x Enterobacter cloacae complex	*27x SARS-CoV-2*
1x Enterobacter hormaechei	
1x Enterococcus malodoratus	
2x Enterococcus faecalis	
21x Escherichia coli	
1x Fusobacterium nucleatum	
1x Haemophilus influenzae	
4x Klebsiella oxytoca	
2x Klebsiella pneumoniae	
1x Providencia rettgeri	
2x Pseudomonas aeruginosa	
1x Streptococcus gordonii	
2x Serratia marcescens	
7x Staphylococcus aureus	
2x Staphylococcus epidermidis	
1x Staphylococcus hominis	
2x Streptococcus anginosus	
1x Streptococcus constellatus	
1x Streptococcus dysgalactiae	
5x Streptococcus pneumoniae	
2x Streptococcus pyogenes	

All pathogens were listed in case of multiple detected pathogens

ESM Table S2: Therapy and complications of bacterial and viral sepsis patients

		Bacterial sepsis (n = 57)	Viral sepsis (n = 33)	p
IMV*		34 (59.6%)	30 (90.9%)	*.002*
NHF in invasively ventilated patients*		13 (22.8%)	17 (51.5%)	*.005*
CPAP in invasively ventilated patients*		7 (12.3%)	13 (39.4%)	*.003*
NHF in not invasively ventilated patients*		5 (8.8%)	3 (9.1%)	*.959*
CPAP in not invasively ventilated patients*		3 (5.3%)	3 (9.1%)	*.483*
Tracheotomy*		5 (9.3%)	14 (46.9%)	*<.001*
Days IMV°		6 (2 – 15)	16 (9 – 23)	*<.001*
Days NHF°		3 (2 – 4)	3 (2 – 8)	*.361*
Days CPAP°		2 (1 – 3)	2 (1 – 7)	*.391*
AKI*		42 (76.4%)	14 (45.2%)	*.004*
	Stage 1	7 (16.7%)	4 (28.6%)	
	Stage 2	15 (35.7%)	4 (28.6%)	*.617*
	Stage 3	20 (47.6%)	6 (42.9%)	
RRT*		28 (49.1%)	11 (34.4%)	*.178*
Days RRT°		6 (4 – 9)	7 (4 – 19)	*.259*
Delay admission-AKI (days)°		0 (0 – 0)	1 (0 – 4)	*.005*
Delay IMV-AKI (days)°		0 (-1 – 0)	1 (0 – 6)	*<.001*
Delay AKI-RRT (days)°		1 (0 – 2)	0 (0 – 1)	*.458*
ECMO*		0	5 (15.6%)	*.002*
Days ECMO°		-	7 (5 – 9)	-
Vasopressors during ICU stay*		57 (100.0%)	29 (90.6%)	*.019*
Length of ICU stay°		6 (3 – 15)	20 (13 – 27)	*<.001*
Length of hospital stay°		20 (9 – 50)	32 (18 – 54)	*.178*

* n (%); ° median (IQR)

IMV – invasive mechanical ventilation; NHF – nasal high flow; CPAP – continuous positive airway pressure; AKI – acute kidney injury; RRT – renal replacement therapy; ECMO – extracorporeal membrane oxygenation.

ESM Table S3: Corticosteroids administered in bacterial and viral sepsis patients

	Bacterial sepsis (n = 57)	Viral sepsis (n = 33)	p
Methylprednisolone*	2 (3.5%)	2 (6.1%)	*.571*
Dexamethasone*	2 (3.5%)	24 (72.7%)	*<.001*
Hydrocortisone*	45 (78.9%)	8 (24.2%)	*<.001*
Other Corticosteroid*	3 (5.3%)	0	*.180*
No Corticosteroid*	11 (19.3%)	1 (3.0%)	*.029*
Days Methylprednisolone°	10 (1 – 19)	7 (5 – 8)	*1*
Days Dexamethasone°	3 (1 – 4)	11 (10 – 17)	*.025*
Days Hydrocortisone°	5 (3 – 8)	7 (3 – 12)	*.566*
Days other corticosteroid°	6 (1 – 7)	-	-

* n (%); ° median (IQR)

ESM Table S4: Adjusted linear regression analysis of factors associated with the maximum IL-6 and PCT levels within 48 hours after ICU admission

**IL-6**	Correlation Coefficient (95% CI)	*p*
Bacterial sepsis	1.093 (0.564 – 1.622)	*< .001*
SAPS 3 at ICU admission	0.021 (-0.002 – 0.044)	*.071*
Sex	-0.234 (-0.706 – 0.238)	*.327*
Site of infection	-0.023 (-0.204 – 0.158)	*.803*
Age	-0.008 (-0.024 – 0.008)	*.307*

**PCT**	Correlation Coefficient (95% CI)	*p*
Bacterial sepsis	1.424 (0.980 – 1.869)	*< .001*
SAPS 3 at ICU admission	0.013 (-0.006 – 0.032)	*.173*
Sex	-0.245 (-0.642 – 0.152)	*.223*
Site of infection	-0.017 (-0.169 – 0.135)	*.823*
Age	-0.001 (-0.014 – 0.013)	*.904*

IL-6 – Interleukin-6; PCT – procalcitonin; SAPS 3 – simplified acute physiology score; ICU – intensive care unit; CI – confidence interval;

ESM Figure S2: Course of inflammatory markers in in SARS-CoV-2-positive patients: comparison between vaccinated and unvaccinated groups

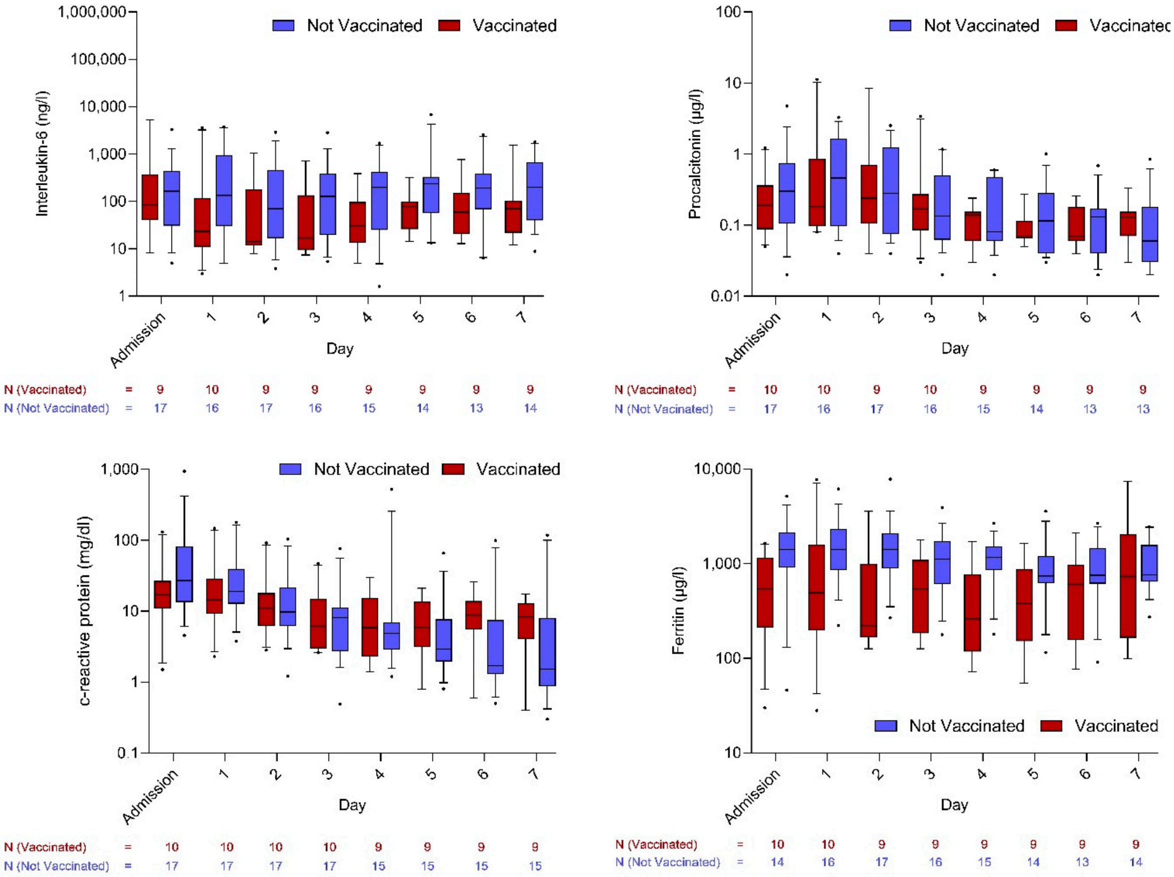

ESM Table S5: Adjusted linear mixed-effects models of associations between infection etiology and inflammatory marker trajectories over time

**IL-6**	Correlation Coefficient (95% CI)	*p*
Bacterial sepsis	372.6 (118.0 – 627.2)	*.004*
SAPS 3 at ICU admission	9.9 (0.4 – 19.3)	*.041*
Sex	219.3 (25.5 – 413.2)	*.027*
Site of infection	-53.3 (-458.4 – 351.7)	*.929*
Age	7.7 (0.5 – 14.8)	*.036*
Hypertension	316.0 (0.5 – 14.8)	*.002*
Coronary artery disease	25.8 (-145.1 – 196.8)	*.766*
Atrial fibrillation	71.6 (-115.8 – 259.0)	*.452*
Chronic obstructive pulmonary disease	66.4 (-251.0 – 383.7)	*.680*
Diabetes mellitus type I	-8483.1 (-48256.9 – 31290.7)	*.673*
Diabetes mellitus type II	115.1 (-79.0 – 309.3)	*.243*
Neurologic comorbidity	520.0 (245.5 – 794.4)	*<.001*
Hepatic comorbidity	-13.3 (-339.8 – 313.3)	*.936*
Pulmonary comorbidity	372.5 (109.3 – 635.7)	*.006*
Chronic kidney failure	257.4 (49.3 – 465.4)	*.016*

**PCT**	Correlation Coefficient (95% CI)	*p*
Bacterial sepsis	-4.5 (-7.6 – -1.4)	*.005*
SAPS 3 at ICU admission	-0.1 (-0.2 – 0.1)	*.313*
Sex	1.2 (-1.1 – 3.5)	*.288*
Site of infection	-0.8 (-4.9 – 3.3)	*.231*
Age	0.1 (-0.0 – 0.2)	*.055*
Hypertension	2.0 (-0.3 – 4.4)	*.090*
Chronic obstructive pulmonary disease	0.5 (-1.5 – 2.6)	*.622*
Atrial fibrillation	-1.6 (-3.8 – 0.7)	*.169*
COPD	-4.8 (-8.5 – -1.1)	*.012*
Diabetes mellitus type I	10.6 (2.8 – 18.5)	*.009*
Diabetes mellitus type II	1.3 (-1.2 – 3.8)	*.293*
Neurologic comorbidity	-1.6 (-5.3 – 2.1)	*.396*
Hepatic comorbidity	2.2 (-2.3 – 6.8)	*.332*
Pulmonary comorbidity	-3.8 (-6.6 – -1.1)	*.008*
Chronic kidney failure	-1.0 (-3.4 – 1.5)	*.440*

**CRP**	Correlation Coefficient (95% CI)	*p*
Bacterial sepsis	-4.0 (-20.7 – 12.7)	*.639*
SAPS 3 at ICU admission	-0.5 (-1.1 – 0.1)	*.115*
Sex	18.0 (5.9 – 30.2)	*.004*
Site of infection	-18.3 (-40.9 – 4.3)	*.005*
Age	0.1 (-0.4 – 0.5)	*.829*
Hypertension	-31.5 (-44.4 – -18.5)	*<.001*
Coronary artery disease	-9.1 (-20.5 – 2.2)	*.113*
Atrial fibrillation	-4.8 (-17.4 – 7.7)	*.449*
Chronic obstructive pulmonary disease	15.3 (-4.9 – 35.4)	*.137*
Diabetes mellitus type I	5.2 (-39.9 – 50.2)	*.821*
Diabetes mellitus type II	35.7 (22.7 – 48.7)	*<.001*
Neurologic comorbidity	0.5 (-18.3 – 19.2)	*.961*
Hepatic comorbidity	14.8 (-7.1 – 36.7)	*.185*
Pulmonary comorbidity	2.5 (-13.6 – 18.6)	*.760*
Chronic kidney failure	-9.0 (-22.7 – 4.8)	*.200*

**Ferritin**	Correlation Coefficient (95% CI)	*p*
Bacterial sepsis	-464.2 (-904.2 – -24.1)	*.039*
SAPS 3 at ICU admission	11.9 (-4.5 – 28.2)	*.153*
Sex	295.6 (-41.6 – 632.8)	*.085*
Site of infection	605.8 (-146.3 – 1357.8)	*.002*
Age	-11.3 (-25.7 – 3.0)	*.121*
Hypertension	-282.5 (-655.7 – 90.9)	*.137*
Coronary artery disease	277.0 (-20.8 – 574.8)	*.068*
Atrial fibrillation	232.1 (-94.3 – 558.6)	*.162*
Chronic obstructive pulmonary disease	788.9 (257.2 – 1320.6)	*.004*
Diabetes mellitus type I	0	
Diabetes mellitus type II	-128.0 (-489.5 – 233.5)	*.485*
Neurologic comorbidity	544.0 (56.6 – 1031.5)	*.029*
Hepatic comorbidity	-16.5 (-599.0 – 566.0)	*.955*
Pulmonary comorbidity	-30.8 (-508.1 – 446.6)	*.899*
Chronic kidney failure	130.4 (-230.1 – 490.8)	*.476*

IL-6 – Interleukin-6; PCT – procalcitonin; CRP – c-reactive protein; SAPS 3 – simplified acute physiology score; ICU – intensive care unit; CI – confidence interval;

ESM Table S6: Adjusted linear mixed-effects model of associations between infection etiology and number of organ dysfunctions

**Organdysfunction**	Correlation Coefficient (95% CI)	*p*
Bacterial sepsis	-0.5 (-0.8 – -0.3)	*<.001*
SAPS 3 at ICU admission	0.0 (0.0 – 0.0)	*<.001*
Sex	0.4 (0.2 – 0.6)	*<.001*
Site of infection	-0.6 (-0.9 – -0.2)	*.017*
Age	-0.0 (-0.0 – -0.0)	*.002*
Hypertension	0.1 (-0.1 – 0.3)	*.357*
Coronary artery disease	-0.2 (-0.4 – 0.0)	*.072*
Atrial fibrillation	-0.5 (-0.7 – -0.3)	*<.001*
Chronic obstructive pulmonary disease	0.1 (-0.3 – 0.4)	*.667*
Diabetes mellitus type I	-0.5 (-1.3 – 0.2)	*.143*
Diabetes mellitus type II	-0.2 (-0.4 – -0.0)	*.031*
Neurologic comorbidity	0.0 (-0.3 – 0.3)	*.844*
Hepatic comorbidity	-0.8 (-1.1 – -0.4)	*<.001*
Pulmonary comorbidity	-0.0 (-0.3 – 0.2)	*.784*
Chronic kidney failure	0.0 (-0.2 – 0.2)	*.926*

SAPS 3 – simplified acute physiology score; ICU – intensive care unit;

ESM Table S7: Doses of norepinephrine and vasopressin per day

	Bacterial sepsis (n = 57)	Viral sepsis (n = 33)	*p*
Max. Norepinephrine dose at day of admission (µg/kg/min)°	0.16 (0.09 – 0.32)	0.07 (0.04 – 0.15)	*.001*
Max. Vasopressin dose at day of admission (U/hr)°	1.60 (1.60 – 2.40)	1.60 (1.60 – 2.40)	*.895*
Max. Norepinephrine dose at day 1 (µg/kg/min)°	0.15 (0.09 – 0.41)	0.05 (0.03 – 0.10)	*<.001*
Max. Vasopressin dose at day 1 (U/hr)°	1.60 (1.60 – 1.60)	1.60 (1.60 – 1.60)	*.760*
Max. Norepinephrine dose at day 2 (µg/kg/min)°	0.07 (0.05 – 0.25)	0.03 (0.01 – 0.06)	*<.001*
Max. Vasopressin dose at day 2 (U/hr)°	1.60 (1.20 – 2.00)	1.60 (1.28 – 1.60)	*.291*
Max. Norepinephrine dose at day 3 (µg/kg/min)°	0.06 (0.03 – 0.16)	0.03 (0.02 – 0.05)	*.009*
Max. Vasopressin dose at day 3 (U/hr)°	1.60 (0.80 – 2.00)	1.60 (0.80 – 1.60)	*.427*
Max. Norepinephrine dose at day 4 (µg/kg/min)°	0.06 (0.04 – 0.13)	0.04 (0.02 – 0.07)	*.045*
Max. Vasopressin dose at day 4 (U/hr)°	1.60 (1.20 – 1.60)	1.60 (0.24 – 1.60)	*.441*
Max. Norepinephrine dose at day 5 (µg/kg/min)°	0.06 (0.04 – 0.09)	0.03 (0.01 – 0.07)	*.193*
Max. Vasopressin dose at day 5 (U/hr)°	1.44 (0.40 – 1.60)	1.20 (0.80 – 1.60)	*.825*
Max. Norepinephrine dose at day 6 (µg/kg/min)°	0.03 (0.02 – 0.06)	0.03 (0.01 – 0.07)	*.914*
Max. Vasopressin dose at day 6 (U/hr)°	0.80 (0.64 – 1.20)	1.60 (1.20 – 1.60)	*.190*
Max. Norepinephrine dose at day 7 (µg/kg/min)°	0.03 (0.02 – 0.06)	0.03 (0.02 – 0.06)	*.595*
Max. Vasopressin dose at day 7 (U/hr)°	2.00 (0.80 – 3.20)	1.40 (1.20 – 1.60)	*1.000*

* n (%); ° median (IQR)

ESM Table S8: Rate of patients fulfilling SEPSIS-3 criteria for sepsis excluding respiratory and cardiovascular SOFA

	Bacterial sepsis (n = 57)	Viral sepsis (n = 33)
Baseline	75.4%	18.2%
Day 1	73.7%	15.2%
Day 2	75.0%	9.1%
Day 3	67.4%	6.1%
Day 4	66.7%	10.0%
Day 5	65.6%	10.3%
Day 6	63.3%	10.3%
Day 7	48.1%	13.8%

Rates are the percentage of patients ≥2 SOFA points if respiratory and cardiovascular section were excluded;

ESM Table S9: Baseline characteristics of the bacterial sepsis group and the viral sepsis group restricted to patients with respiratory infection

		Bacterial sepsis (n = 15)	Viral sepsis (n = 33)	*p*
Age°		64 (56 – 75)	66 (51 – 71)	*.169*
Sex (male)*		11 (73.3%)	25 (75.8%)	*.857*
BMI°		25.4 (23.5 – 26.6)	28.1 (23.2 – 30.9)	*.859*
HBA1c%°		5.6 (5.1 – 5.8)	6.0 (5.5 – 6.4)	*.213*
SOFA at ICU admission°		9 (8 – 11)	7 (6 – 8)	*.018*
SAPS 3 at ICU admission°		70 (63 – 78)	57 (52 – 67)	*.033*
Hospital admission due to sepsis symptoms*		9 (60.0%)	2 (6.7%)	*<.001*
Risk factor*	Trauma	0	1 (3.0%)	*.081*
	Surgery	1 (6.7%)	0	
	Sepsis in PMH	3 (20.0%)	1 (3.0%)	
	No risk factor	11 (73.3%)	31 (93.9%)	
SIRS criteria fulfilled at ICU admission*		11 (73.3%)	13 (40.6%)	*.037*
Vasopressors at ICU admission*		15 (100.0%)	28 (84.8%)	*.111*
Norepinephrine dose at ICU admission (µg/kg/min)°		0.17 (0.08 – 0.46)	0.07 (0.04 – 0.15)	*.197*
Vasopressin dose at ICU admission (U/h)°		1.60 (1.60 – 2.40)	1.60 (1.60 – 2.40)	*.707*
**Inflammatory markers at ICU admission**				
Interleukin-6 (ng/l)°		2513.00 (1678.00 – 7484.00)	165.00 (39.10 – 460.20)	*<.001*
Procalcitonin (µg/l)°		11.60 (2.82 – 31.46)	0.28 (0.10 – 0.55)	*<.001*
C-reactive protein (mg/dl)°		24.40 (11.54 – 41.10)	19.50 (12.34 – 34.90)	*.755*
Ferritin (µG/l)°		378.10 (313.00 – 479.00)	951.00 (325.00 – 1640.00)	*.420*
**Comorbidities**				
Hypertension*		8 (53.3%)	11 (33.3%)	*.189*
Coronary artery disease*		7 (46.7%)	8 (24.2%)	*.120*
Atrial fibrillation*		5 (33.3%)	7 (21.2%)	*.369*
COPD*		6 (40.0%)	1 (3.0%)	*<.001*
Diabetes mellitus type I*		1 (6.7%)	0	*.134*
Diabetes mellitus type II*		3 (20.0%)	7 (21.2%)	*.924*
Neurologic comorbidity*		0	5 (15.2%)	*.111*
Hepatic comorbidity*		2 (13.3%)	0	*.032*
Pulmonary comorbidity*		1 (6.7%)	4 (12.1%)	*.566*
Chronic kidney failure*		1 (6.7%)	9 (27.3%)	*.103*
ICU mortality*		6 (40.0%)	4 (12.1%)	*.027*
Hospital mortality*		6 (40.0%)	5 (15.2%)	*.058*
28-Day mortality*		6 (40.0%)	4 (12.1%)	*.027*
Standardised mortality rate (SOFA)		1.20	0.56	-
Standardised mortality rate (SAPS 3)		0.70	0.40	-

* n (%); ° median (IQR)

ICU – intensive care unit; SOFA – sequential organ failure assessment; SAPS 3 – simplified acute physiology score; PMH – past medical history; SIRS – systemic inflammatory response syndrome; COPD – chronic obstructive pulmonary disease. SIRS criteria were fulfilled ≥2 points.

ESM Table S10: Therapy and Outcome of bacterial and viral sepsis patients restricted to patients with respiratory infection

		Bacterial sepsis (n = 15)	Viral sepsis (n = 33)	*p*
IMV*		11 (73.3%)	30 (90.9%)	*.110*
NHF in invasively ventilated patients*		4 (26.7%)	17 (51.5%)	*.108*
CPAP in invasively ventilated patients*		4 (26.7%)	13 (39.4%)	*.393*
NHF in not invasively ventilated patients*		1 (6.7%)	3 (9.1%)	*.778*
CPAP in not invasively ventilated patients*		1 (6.7%)	3 (9.1%)	*.778*
Tracheotomy*		3 (21.4%)	14 (46.9%)	*.104*
Days IMV°		15 (10 – 20)	16 (9 – 23)	*.207*
Days NHF°		3 (2 – 4)	3 (2 – 8)	*.262*
Days CPAP°		2 (1 – 2)	2 (1 – 7)	*.561*
AKI*		11 (78.6%)	14 (45.2%)	*.037*
	Stage 1	2 (18.2%)	4 (28.6%)	
	Stage 2	4 (36.4%)	4 (28.6%)	*.817*
	Stage 3	5 (45.5%)	6 (42.9%)	
RRT*		7 (46.7%)	11 (34.4%)	*.419*
Days RRT°		8 (2 – 16)	7 (4 – 19)	*.534*
Delay admission-AKI (days)°		0 (0 – 1)	1 (0 – 4)	*.341*
Delay IMV-AKI (days)°		0 (0 – 0)	1 (0 – 6)	*.081*
Delay AKI-RRT (days)°		1 (1 – 1)	0 (0 – 1)	*.732*
ECMO*		0	5 (15.6%)	*.105*
Days ECMO°		-	7 (5 – 9)	-
Vasopressors during ICU stay*		15 (100.0%)	29 (90.6%)	*.220*
Length of ICU stay°		16 (3 – 23)	20 (13 – 27)	*.238*
Length of hospital stay°		23 (7 – 59)	32 (18 – 54)	*.507*

* n (%); ° median (IQR)

IMV – invasive mechanical ventilation; NHF – nasal high flow; CPAP – continuous positive airway pressure; AKI – acute kidney injury; RRT – renal replacement therapy; ECMO – extracorporeal membrane oxygenation.

ESM Figure S3: Course of inflammatory markers in comparison between bacterial and viral sepsis patients restricted to patients with respiratory infection

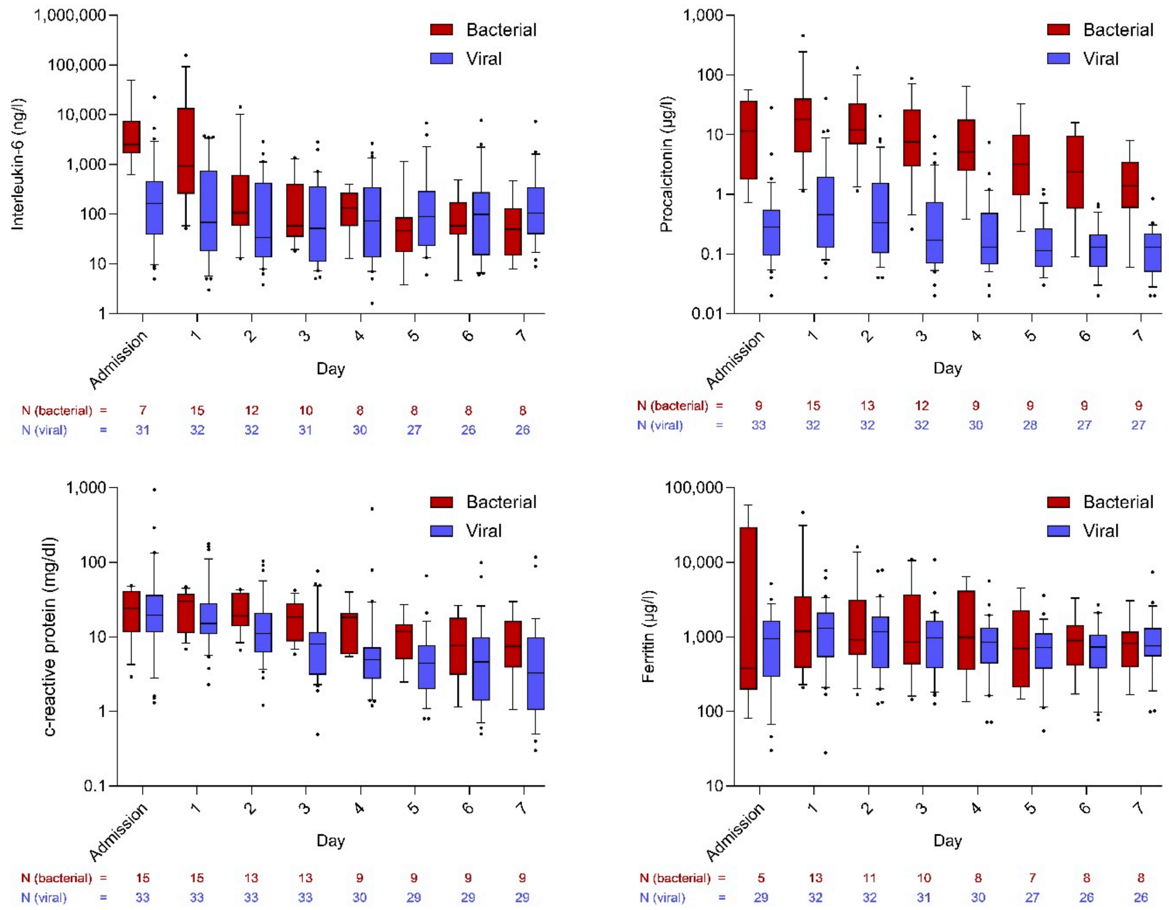

ESM Table S11: Adjusted linear mixed-effects models of associations between infection etiology and inflammatory marker trajectories over time restricted to patients with respiratory infection

**IL-6**	Correlation Coefficient (95% CI)	*p*
Bacterial sepsis	547.5 (160.0 – 934.9)	*.006*
SAPS 3 at ICU admission	20.8 (4.9 – 36.6)	*.011*
Sex	210.3 (-109.1 – 529.7)	*.194*
Age	6.7 (-3.8 – 17.2)	*.207*
Hypertension	368.6 (51.2 – 685.9)	*.023*
Coronary artery disease	-3.1 (-292.8 – 286.7)	*.983*
Atrial fibrillation	89.9 (-214.0 – 393.8)	*.559*
Chronic obstructive pulmonary disease	215.6 (-276.7 – 708.0)	*.387*
Diabetes mellitus type I	-8877.1 (-59039.2 – 41285.1)	*.723*
Diabetes mellitus type II	148.0 (-214.4 – 510.4)	*.420*
Neurologic comorbidity	613.2 (236.1 – 990.3)	*.002*
Hepatic comorbidity	-333.3 (-1023.0 – 356.4)	*.340*
Pulmonary comorbidity	471.2 (65.0 – 877.4)	*.023*
Chronic kidney failure	350.9 (31.6 – 670.1)	*.032*

**PCT**	Correlation Coefficient (95% CI)	*p*
Bacterial sepsis	-3.2 (-5.1 – -1.3)	*.001*
SAPS 3 at ICU admission	0.0 (-0.0 – 0.1)	*.369*
Sex	2.4 (0.9 – 3.9)	*.002*
Age	-0.0 (-0.0 – 0.0)	*.234*
Hypertension	-3.3 (-4.8 – -1.8)	*<.001*
Coronary artery disease	0.5 (-0.8 – 1.9)	*.418*
Atrial fibrillation	-2.1 (-3.4 – -0.7)	*.005*
Chronic obstructive pulmonary disease	-3.4 (-5.6 – -1.3)	*.003*
Diabetes mellitus type I	7.9 (3.7 – 12.2)	*.001*
Diabetes mellitus type II	1.8 (0.0 – 3.5)	*.046*
Neurologic comorbidity	0.0 (-1.8 – 1.8)	*.968*
Hepatic comorbidity	1.2 (-2.3 – 4.6)	*.501*
Pulmonary comorbidity	1.3 (-0.5 – 3.1)	*.149*
Chronic kidney failure	-0.7 (-2.0 – 0.7)	*.316*

**CRP**	Correlation Coefficient (95% CI)	*p*
Bacterial sepsis	-8.6 (-16.8 – -0.5)	*.037*
SAPS 3 at ICU admission	-0.1 (-0.4 – 0.2)	*.522*
Sex	-1.2 (-7.7 – 5.2)	*.708*
Age	-0.1 (-0.3 – 0.1)	*.421*
Hypertension	-5.3 (-12.2 – 1.6)	*.134*
Coronary artery disease	2.3 (-3.8 – 8.5)	*.455*
Atrial fibrillation	0.0 (-6.5 – 6.5)	*1.000*
Chronic obstructive pulmonary disease	-3.3 (-13.3 – 6.7)	*.511*
Diabetes mellitus type I	16.9 (-3.0 – 36.8)	*.096*
Diabetes mellitus type II	6.6 (-0.9 – 14.1)	*.086*
Neurologic comorbidity	-4.2 (-12.0 – 3.7)	*.295*
Hepatic comorbidity	8.3 (-6.5 – 23.0)	*.270*
Pulmonary comorbidity	-22.0 (-30.5 – -13.4)	*<.001*
Chronic kidney failure	-3.2 (-9.5 – 3.1)	*.320*

**Ferritin**	Correlation Coefficient (95% CI)	*p*
Bacterial sepsis	-257.6 (-888.2 – 373.0)	*.420*
SAPS 3 at ICU admission	35.8 (10.5 – 61.1)	*.006*
Sex	650.8 (153.1 – 1148.4)	*.011*
Age	-20.2 (-38.2 – -2.1)	*.029*
Hypertension	-644.5 (-1160.8 – -128.2)	*.015*
Coronary artery disease	142.0 (-306.4 – 590.4)	*.532*
Atrial fibrillation	293.5 (-170.2 – 757.2)	*.212*
Chronic obstructive pulmonary disease	1715.3 (985.2 – 2445.4)	*<.001*
Diabetes mellitus type I	0	
Diabetes mellitus type II	-91.7 (-666.3 – 482.8)	*.752*
Neurologic comorbidity	723.9 (136.2 – 1311.5)	*.016*
Hepatic comorbidity	-741.2 (-1876.4 – 393.9)	*.199*
Pulmonary comorbidity	444.3 (-162.6 – 1051.1)	*.150*
Chronic kidney failure	124.1 (-345.3 – 593.5)	*.601*

IL-6 – Interleukin-6; PCT – procalcitonin; CRP – c-reactive protein; SAPS 3 – simplified acute physiology score; ICU – intensive care unit; CI – confidence interval;

ESM Table S12: Rate of patients fulfilling SEPSIS-3 criteria for sepsis excluding respiratory and cardiovascular SOFA restricted to patients with respiratory infection

	Bacterial sepsis (n = 57)	Viral sepsis (n = 33)
Baseline	66.7%	18.2%
Day 1	46.7%	15.2%
Day 2	69.2%	9.1%
Day 3	53.8%	6.1%
Day 4	55.6%	10.0%
Day 5	55.6%	10.3%
Day 6	44.4%	10.3%
Day 7	44.4%	13.8%

Rates are the percentage of patients ≥2 SOFA points if respiratory and cardiovascular section were excluded
